# Anderson Localized Plasmon in Graphene with Random Tensile‐Strain Distribution

**DOI:** 10.1002/advs.201801974

**Published:** 2019-02-06

**Authors:** Jiahua Duan, Sanshui Xiao, Jianing Chen

**Affiliations:** ^1^ Institute of Physics Chinese Academy of Sciences 100190 Beijing China; ^2^ School of Physical Sciences University of Chinese Academy of Sciences 100049 Beijing China; ^3^ DTU Fotonik Department of Photonics Engineering and Center for Nanostructured Graphene Technical University of Denmark DK2800 Kgs. Lyngby Denmark; ^4^ Beijing National Laboratory for Condensed Matter Physics 100190 Beijing China; ^5^ Songshan Lake Materials Laboratory Dongguan 523808 Guangdong China

**Keywords:** disorder, graphene plasmons, strains, strong localization

## Abstract

Anderson localization, the unusual phenomenon discovered in a disordered medium, describes the phase transition from the extended to localized state. Owing to the interference in multiple elastic scattering, this concept is firstly demonstrated in an electron system, then to photon and matter waves. However, Anderson localization has not been observed for polaritonic waves with its unique features of strong field confinement and tunability. Here, Anderson localization of plasmon polaritons is experimentally reported in a flat graphene sheet simultaneously with homogenous charge carrier and random tensile‐strain distributions. By selectively choosing different disordered levels, the transition from quasi‐expansion to weak localization, and finally Anderson localization are observed. Relying on the infrared nanoimaging technique, the spatial dependence of the localization is further studied, and finally the transition window from weak to Anderson localization of graphene plasmon polaritons is identified with the aid of the scaling theory. The experimental approach paves a new way to study Anderson localization in other polaritonic systems such as phonon, exciton, magnon polaritons, etc.

The phase transition from ballistic state to localization in a disordered medium is first predicted by Anderson.^1^ With increasing the disorder, the constructive interference of certain scattering paths and coherence induce weak localization, and then, Anderson localization.^2^ Due to strong electron–electron interaction, Anderson localization of electrons can be only indirectly identified by the deduction in the electronic transportation measurements^3^, ^4^ including hopping conductivity and positive magneto‐conductivity. While, for Boson‐type electromagnetic^5^, ^6^, ^7^, ^8^ and matter waves,^9^, ^10^ Anderson localization has been directly observed and theoretically predicted^11^ ascribed to their noninteraction and natural coherence. Polaritons,^12^, ^13^, ^14^, ^15^ coupling photons with other particles such as electrons, phonons, excitons, and their hybrids, are also one type of Bosonic wave providing strong field confinement^16^, ^17^, ^18^ and nanoscale manipulation of photons.^19^, ^20^ The theoretical model^21^ has predicted that the Anderson localization of polaritons exists in the 2D metal film with high disordered level. When the Anderson localization occurs, the polaritonic distribution is localized within localization length (ξ) and its enhanced near‐field amplitude (*E*_max_) is inversely proportional to the ξ, as *E*_max _ ≈ *E*_0_ε′/ε′′(*a*/ξ)^2^ (*a* is a structure parameter, ε′ and ε′′ are the real‐ and imaginary‐part of the material dielectric constant). However, the experimental observation of Anderson localized polaritons remains challenging, because of hyperdispersion of polaritonic signal itself caused by rough morphology and electric heterogeneity. Compared with the case for metal, graphene^22^, ^23^ with atomically flat surface and lower carrier density (controllable) provide an applicable platform for studying Anderson localization of plasmon polaritons. Compared with Fermi wavevector of electrons in metal, smaller wavevector of graphene plasmon^23^ also makes its Anderson localization more easily observable.

In this communication, we report Anderson localization of plasmon polaritons in flat graphene flakes simultaneously having randomly distributed tensile‐strains and homogeneous charge carrier. By carefully selecting graphene samples with different levels of disorder, we observe the transition for plasmon polaritons from quasi‐expansion, weak localization, to Anderson localization. With the aid of scaling theory, we identify the criterion for the transition from weak to Anderson localization. Using nanoinfrared imaging technique, we analyze the properties of graphene plasmon in different states. These experimental results shed light on studying the Anderson localization of various polaritons and provide a new freedom for manipulating graphene plasmons.

Due to good flexibility of single‐layer graphene, the mechanically exfoliated process is similar to throw a soft handkerchief on stiff surface, resulting in inhomogeneous strain. The thermal treatment during fabrication can also give rise to roughness of strain due to different thermal expansion efficiencies of graphene and substrate. It is worth noting that the spatial distribution of strains and carrier density varies from sample to sample. By preparing hundreds of graphene samples, we characterize the distribution of strains and doping level by Raman spectroscopy^24^ and select three representative flakes with specific disordered level and similar doping level (homogenously distributed). The disordered level of graphene is quantified by:

(1) $$\Delta \left(\%\right)\;=\left(\rho_{\max}-\rho_{\min}\right)/\left(\rho_{\max}+\rho_{\min}\right)$$

Here, ρ_max_ and ρ_min_ represent the largest and smallest value in strain map, respectively. **Figure** **1**a,b show the schematic diagram of graphene plasmons in ordered and disordered systems, respectively, where the plasmons are launched and probed by scattering‐type scanning near‐field optical microscopy (s‐SNOM).^19^, ^20^, ^25^, ^26^ By correlative analysis of the Raman G and 2D modes, the native strain can be unambiguously measured in spite of the interference from the coexisted hole‐doping effect from the substrate. As shown in Figure 1c–e, we show the Raman maps of different graphene flakes with disordered level of ≈0%, ≈18.2%, and ≈31.3%, respectively. The red‐dashed lines in Figure 1 mark the physical edges of graphene. In order to characterize the spatial components of random disorder and extract its characteristic length scale, we also conduct the Fourier transform of strain maps and measure the size of every strain spot. For the medium (≈18.2%) and high (≈31.3%) disorder level, the length scale of disorder is 263 ± 101.4 and 304 ± 170.3 nm, which are both in the same order of magnitude with plasmonic wavelength (≈220 nm) in graphene. Besides the Anderson localization, there are two additional factors resulting in the enhanced field amplitude and localization of graphene plasmons: surface roughness and inhomogeneous carrier concentration.^27^ In order to avoid the adverse impact caused by these two factors, we carefully select representative graphene samples with atomically flat surface and homogeneously distributed carrier concentration. As shown in Figure 1f,g, our three samples have homogenously spatially distributed carrier density and similar doping level (≈ 5  × 10^12^cm^−3^). This homogenous doping level and atomically flat graphene surface are necessary for the observation of Anderson localization.

**Figure 1:**
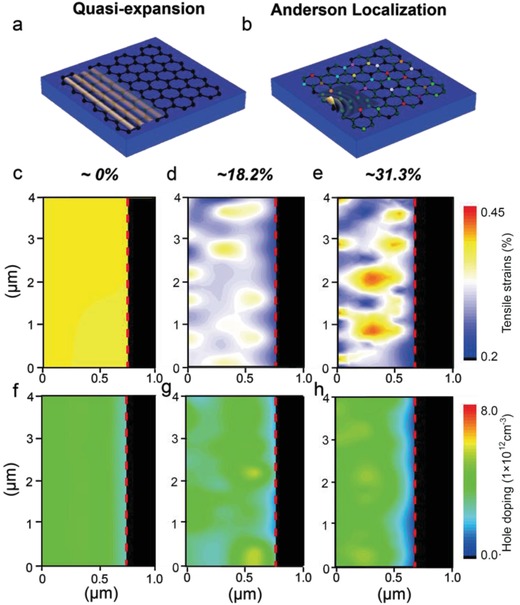
a,b) Schematic diagrams of graphene plasmon in ordered and disordered graphene. The different colors in (b) represent the carbon atoms with different strains, indicating the disorder caused by randomly distributed strains. c–e) Raman maps of tensile‐strain distribution in ordered, weak disordered, and strong disordered graphene systems. f–h) Spatial distribution of charge carrier shows similarly homogeneous in ordered, weak disordered, and strong disordered systems. Meanwhile, the doping levels in three conditions are similar, as ≈5 × 10^12^ cm^−3^. Graphene edge is marked with the red dashed line.

For a 2D system, the scaling theory^28^ predicts that there are no actual extended states and that the localization always occurs for any amount of disorder (unlike a 3D system, where localization occurs above one critical disordered level). However, the wave in the 2D system would be only marginally localized with rather large localization length (≈infinite) and in a quasi‐extended state^2^ when the system is weak disordered. With medium disorder, the weak localization takes place. Then, the wave should display a crossover, from weak localization to Anderson localization, as the disorder level is further increased. In order to experimentally observe Anderson transition, we record the fourth‐order demodulated harmonics of near‐field amplitude of graphene plasmons with the help of s‐SNOM. Representative near‐field images of three graphene systems with different disordered levels mentioned above are shown in **Figure** **2**a–c. In all three conditions, one of the most distinct features of graphene plasmon is the presence of fringes damply propagating along the *Y*‐direction^20^ (the green arrows in Figure 2a–c). In the 2D system, Anderson localization exists in both *X*‐ and *Y*‐direction. However, it is more difficult to distinguish Anderson localization from inherent damping property along the *Y*‐direction, compared with *X*‐direction. In order to extract the localization length, we evaluate the average intensity ($〈|E(x){|}^{2}〉\;\;=\int \frac{〈|E(x,y){|}^{2}〉}{L_{y}}\;dy$, *L_y_* denotes the decay length of graphene plasmon in the *Y*‐direction), and the corresponding results are shown as black dot‐lines in Figure 2d–f. We use the common criterion^5^, ^6^, ^7^, ^9^, ^10^ to distinguish weak and Anderson localization, i.e., Gaussian fitting in both linear and semi‐log coordinates for the weak scattering, and exponential fitting in linear coordinate and linear fitting in semi‐log coordinate for the Anderson condition.^29^ Based on the reported routine,^5^ the localization length for weak and Anderson condition is obtained by Gaussian ($\exp\;(-\frac{2x^{2}}{\xi^{2}})$) and exponential (exp(−2∣x∣/ξ)) fitting. When the graphene lattice is almost periodic (Δ ≈ 0%), the plasmon undergoes quasi‐extended transport with the localization length (ξ) close to infinite, manifested by almost uniform average field intensity in Figure 2d. For the case of graphene with the disordered level of 18.2%, it is better fitting with Gaussian shape both in linear and semi‐log coordinates; see the blue solid lines in Figure 2e.

**Figure 2:**
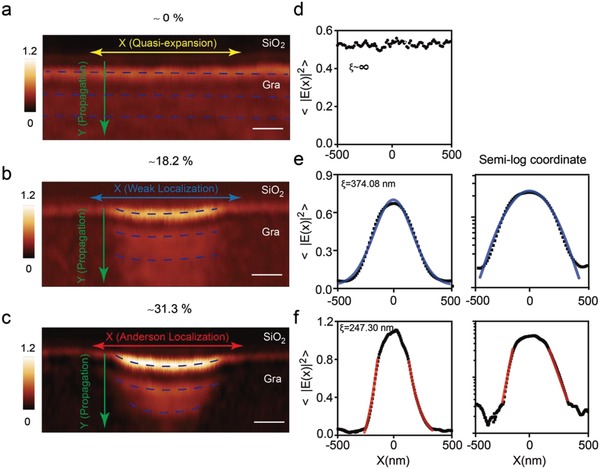
a–c) The near‐field images of three transport patterns (at the incident frequency of ω_0_ = 901 cm^−1^) including quasi‐expansion, weak localization, and Anderson localization. The blue‐dashed lines guide for the plasmonic fringes. d–f) The average intensity along the *X*‐direction, see the black dots, for these three conditions in linear coordinates. In the right panels of (e) and (f), we represent the results in semi‐log coordinates. The blue lines in (e) are obtained by Gaussian fitting to the intensity distribution. The red lines in the left panel of (f) are associated with exponential fits to the wings, and the right panel for linear fitting. Scale bars, 300 nm.

Based on the criterion mentioned above, we conclude that plasmon exhibits the weak localization in this disordered level. When Δ further increases to 31.3%, we confirm Anderson localization (Figure 2f) of graphene plasmon by the criterion above. The localization length (≈247.30 nm) in Anderson condition is much smaller than that in the weak scattering case (≈374.08 nm). The transition from Figure 2b,c illustrates the crossover from the weak to Anderson localization along with increasing of the disordered level. The Anderson localization observed here is mainly caused by the disorder of the tensile‐strain, instead of the inhomogeneous carrier density, see Figure 1. We further evaluate the field enhancement factor (defined by ratio of the maximum value of average intensity with respect to that for the expansion case), which is ≈1.4 and ≈2 for the weak and Anderson case, respectively. The result observed here is consistent with enhanced light‐matter interaction caused by Anderson localization.^30^, ^31^, ^32^, ^33^ It has potential to further enhance field intensity in cryogenic environment because of suppression of high loss of plasmons caused by multiple scattering of Dirac electrons and small mean‐free‐path at ambient temperature. The Anderson localization paves a new way to enhance light‐matter interaction that is important for the future polaritonic devices.

In order to study intrinsic properties of plasmonic modes for different disordered graphene, we show the line‐profiles of average field intensity along vertical edge direction (*Y*‐direction) at the incident frequency of 901 cm^−1^ in **Figure** **3**a–c. Given the interference between tip‐launching and edge‐reflecting plasmon, we extract properties of graphene plasmon from near‐field images, including wavelength, damping rate, and dispersion.^19^, ^20^, ^34^ Given that the percentage of changing strain is in the same order of magnitude with the change of plasmonic wavelength, the modest strain (<0.45%) in our case only changes the plasmon wavelength by less than 1%. We find that the plasmonic wavelength is similar, i.e., λ_p_ ≈ 216.7 nm for the quasi‐expansion, λ_p_ ≈ 206.7 nm for the weak localization and λ_p_ ≈ 220.0 nm for the Anderson localization. The similarity is mainly arisen from same doping level (or Fermi level) for these three samples. The plasmonic damping increases when Anderson transition occurs, i.e., γ ≈ 0.20 for the quasi‐expansion, γ ≈ 0.24 for the weak localization and γ ≈ 0.26 for the Anderson localization. By choosing different working frequencies for these samples, we further study how the dispersion of graphene plasmon depends on the localization state see Figure 3d. The background color in Figure 3d shows the theoretical calculation of imaginary part of the Fresnel reflection coefficient. For all selective working frequencies, the experimental results (symbols) for these three different states agree well with the calculated dispersion relation, indicating that the Anderson transition has weak influence on the dispersion of graphene plasmon. One of the key advantages of polaritonics is to break the diffraction limit (≈λ_0_/2) and to confine light on nanoscale. The Y‐confinement factor (λ_0_/λ_p_ ∈ (40,  60)) remains almost unchanged for all three modes. However, the confinement of light in the *X*‐direction is realized through the mode localization arisen from the disordered system. As shown in Figure 3e, the X‐confinement factor (λ_0_/ξ) increases from zero, ≈30, and finally ≈40, when the state changes from quasi‐expansion, weak localization to Anderson localization.

**Figure 3:**
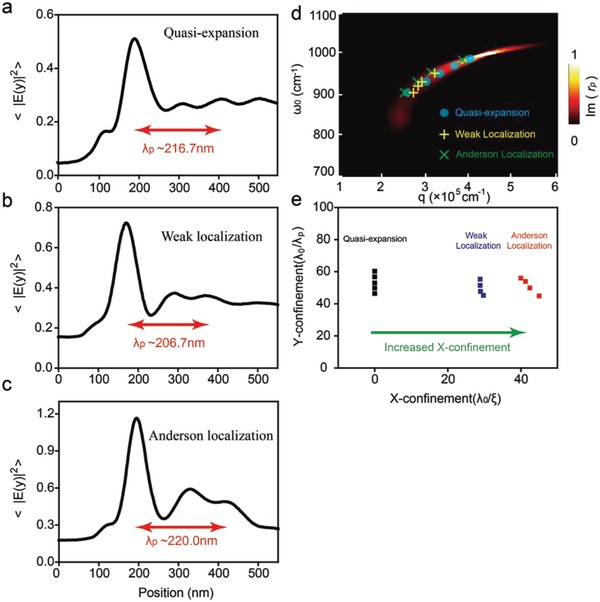
a–c) The average intensity along the *Y*‐direction for quasi‐expanded, weak localized and Anderson localized status. The red arrows denote the wavelength of graphene plasmons. d) The dispersion relation of graphene plasmon for these three states. The scattered points show the extracted experimental values and the background color shows the imaginary part of the Fresnel reflection coefficient, indicating that the Anderson transition has little influence on dispersion of graphene plasmon. e) Extracted field confinement factor. The X‐confinement factor increases during Anderson transition while keeping same for the Y‐confinement.

In **Figure** **4**a, we plot the localization length in both weak and strong localized regime as a function of incident frequency. The black squares and red circles are extracted from near‐field images with Δ ≈ 18.2% and Δ ≈ 31.3%, respectively. The error bars show the standard deviation from data analysis process. We find that the localization length is nearly independent to the frequency. Given of similar and homogenously‐distributed doping levels in two samples, the Anderson transition is an inherent phenomenon only related to the disordered level. In a random system, the scaling theory^7^, ^30^ (ξ = exp(0.5*πk*_p_ l*), where *k*_p_ and l* are wavevector and mean free path of photon, respectively) and Ioffe–Regel criterion^35^ (*k*_p_ l* ≈ 1) are commonly used to analyze the scattering behavior. Taking the photonics‐polaritonics analogy^32^ into account, we apply these theories to analyze the Anderson localization of graphene plasmon in 2D disordered systems.

**Figure 4:**
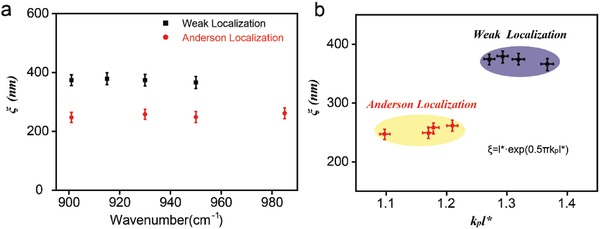
a) The localization length as a function of incident frequency for the weak and Anderson localized states. The error bars are statistical standard deviations with multiple measurements. b) Localization length versus *k*_p_ l* for weak and strong localization regions. The *k*_p_ and l* represent the wavevector and mean free path of graphene plasmons, respectively. The l* is calculated by the scaling theory (inset in Figure 4b).

We evaluate l* and *k*_p_ l* in both the weak and the strong localization. In a pristine graphene flake, the plasmonic mean free path (l* ≈ 1 µm) is much larger than its wavelength (≈200 nm) and *k*_p_ l* ≈ 30 is much larger than one.^23^ In this condition, the transport behavior can be described well by classical theory, showing the expansion state with minimal scatterings. The mean free path in Anderson localization is smaller than that in the weak localization. As an example, for the frequency of 901 cm^−1^, l* is 50.8 nm for the weak condition and 44.1 nm for the strong condition. The strong multiple scattering induces Anderson localization. Also, the observed Anderson transition indicates that the intravalley elastic scattering occurs much more often than the intervalley scattering in the disordered graphene system.^23^ Figure 4b shows the relation between the localization length and scattering strength (*k*_p_ l*). Our scattering strength (1.1–1.2) for the Anderson localization case is close to the Ioffe–Regel criterion^35^ (*k*_p_ l* ≈ 1), while a bit far away for the weak localization (>1.3). This measurement from Anderson to weak localization allows us to identify the Anderson transition window for the graphene plasmons.

In summary, we experimentally observe Anderson localization of graphene plasmon polaritons and study the corresponding properties in 2D disordered systems. Noting that the doping levels in three samples are similar and homogenously spatially distributed, the Anderson localization of plasmons addressed here is mainly caused by random distribution of tensile‐strains. We apply s‐SNOM to directly access the localization length in both weak (ξ ≈ 374.08 nm) and strong (ξ ≈ 247.30 nm) scattering regime. The Anderson localized mode exhibits enhanced near‐field amplitude and strong field confinement. The localization has weak influence on the intrinsic properties of plasmons. Based on the scaling theory, we show the transition window from the weak to Anderson localization of graphene plasmons. Our findings pave a promising way to study polaritonic phenomena in disordered system and to stimulate further work in random polaritonics field.

## Experimental Section

*Sample Preparation and Characterization*: Microcrystals of graphene are mechanically exfoliated from bulk samples and then transferred to 285 nm thick SiO_2_/Si substrates. To get rid of the air between graphene and substrate and to induce strains, thermal treatment (90 °C for 20 min) was conducted on fabricated samples. The monolayer graphene was further identified by optical measurement and Raman Spectroscopy. The surface roughness of graphene was characterized by atomic force microscopy (AFM), as shown in Figure S7 in the Supporting Information. By analyzing the shift of Raman peaks (G/2D), the spatial distribution of tensile‐train and doping level in graphene flakes was obtained. It is worth noting that the mechanical exfoliation was relatively a random process and the fabrication varied sample to sample. Three different disordered graphene samples were selected with similar and homogenous carrier density over hundreds of preparations.

*Infrared s‐SNOM Measurements*: The nanoimaging experiments were performed using an s‐SNOM. The s‐SNOM was a commercial system (Neaspec GmbH) based on an AFM operating in the tapping mode with Ω ≈ 300 kHz and an amplitude of ≈30 nm. The incident frequency spanned from 901 to 980 cm^−1^. A pseudo‐heterodyne interferometric method was applied to extract both the near‐field amplitude and phase of graphene plasmons. The near‐field amplitude was normalized as:$s_{4}\;(\omega )=s_{4}^{0}\;(\omega ){\mathrm{/s}}_{4}^{\mathrm{Si}}(\omega )$. Here, $s_{4}^{0}(\omega )$ and $s_{4}^{\mathrm{Si}}(\omega )$ are the forth‐order demodulated harmonics of the near‐field amplitude detected for graphene and Si standard reference samples, respectively. All nanoIR images were collected at ambient atmosphere.

*Theoretical Calculation of the Dispersion Diagram*: In order to get the dispersion, the complex reflectivity *r_p_* (*q*, ω) of graphene/SiO_2_ structure was calculated. The reflectivity is expressed as^36^:

(2) $$r_{p}=\frac{\varepsilon_{2}k_{1z}-\varepsilon_{1}k_{2z}+k_{1z}k_{2z}\sigma \left(q,\omega \right)/\left(\varepsilon_{0}\omega \right)}{\varepsilon_{2}k_{1z}+\varepsilon_{1}k_{2z}+k_{1z}k_{2z}\sigma \left(q,\omega \right)/\left(\varepsilon_{0}\omega \right)}$$

where ε_0_ is the vacuum permittivity, ε_1_ and ε_2_ are relative permittivity of air and SiO_2_. ω is incident frequency and *q* is the plasmonic wavevector, respectively. The *k*_1*z*_ and *k*_2*z*_ represent the *z*‐components of the wavevector of the incident and the transmitted plane‐waves, respectively. The conductivity of graphene (σ (*q*,  ω)) was derived with random‐phase‐approximation (RPA) with the local limit. The Fermi level was extracted from Raman spectrum.

## Conflict of Interest

The authors declare no conflict of interest.

## Supporting information

Supplementary
