# Supplementary material for: Anderson Localized Plasmon in Graphene with Random Tensile‐Strain Distribution
Source: Adv Sci (Weinh). 2019 Feb 6;6(7):1801974. doi: 10.1002/advs.201801974 (PMC6446603; doi:10.1002/advs.201801974)
Supplement: Supplementary file 1 — Supplementary [file ADVS-6-1801974-s001.pdf]

Copyright WILEY-VCH Verlag GmbH & Co. KGaA, 69469 Weinheim, Germany, 2019.

ADVANCED SCIENCE
Open Access

Supporting Information

for *Adv. Sci.,* DOI: 10.1002/advs.201801974

Anderson Localized Plasmon in Graphene with Random Tensile-Strain Distribution

Jiahua Duan, Sanshui Xiao, and Jianing Chen**

Copyright WILEY-VCH Verlag GmbH & Co. KGaA, 69469 Weinheim, Germany, 2019.

Supporting Information

Anderson localized plasmon in graphene with random tensile-strain distribution

Jiahua Duan, Sanshui Xiao, Jianing Chen**

1. **Optical and Raman characterization of graphene flakes**
2. **Determination of graphene edge through Raman Spectrum**
3. **Separation of mechanical strains from charge doping in graphene**
4. **Extraction of characteristic length scale of the disorder**
5. **Determination of surface roughness of graphene flakes**
6. **Nano-infrared images of graphene plasmon with different localized state**
7. **Fitting method of the damping rate of graphene plasmon**
8. **Calculation of mean free paths of graphene plasmons**
9. **Theoretical calculation of dispersion of graphene plasmons**
10. **The theory of Anderson localization in a 2D metal system**

1.

Optical and Raman characterization of graphene flakes

Our microcrystals of graphene are mechanically exfoliated from bulk graphite samples and then transferred to 285-nm thick SiO_2/Si substrate. The fabricated single-layered graphene can be easily distinguished from multi-layered samples through optical microscopy due to its low optical contrast. In Fig.S1, we show the optical images of graphene flakes with different disordered level. No matter how the strains distribute, the monolayer graphene shows similar optical contrast. The size of our graphene flakes is dozens of square microns.

In order to guarantee the high-quality of single-layered graphene, we conduct the Raman measurement on every fabricated samples. In Fig.S2, we show the representative Raman spectrum collected in central area of graphene flake. There are four distinctive Raman responses in our monolayer graphene: (1) The ratio between intensity of 2D and G band is about two; (2) The bandwidth of Raman band is narrow (FWHM ~28 cm^{-1} for 2D peak and FWHM ~9 cm^{-1} for G peak); (3) The defect-related D band at ~1350 cm^{-1} is absent; (4) The 2D band is great fitted by single Lorentz function. All these Raman properties show indisputable signs of our single-layered graphene without defects[1]. It is worth noting that graphene is easily contaminated under ambient atmosphere and the freshly fabricated samples are optimal candidate for the near-field measurements.

2. Determination of graphene edge through Raman Spectrum

The so-called D band is related to the breathing mode of sp^2 rings and requires a defect to be activated[2]. In our mechanically exfoliated graphene flakes, the D peak is usually unobservable due to few structure defects. However, the D band is seen at graphene edges (Fig.S3a), which act as one-dimensional defects allowing elastic backscattering of electrons. Based on that phenomenon, we can determine the position of graphene edges through Raman line-mapping along the vertical direction of edge[2]. When the incident laser spot moves from substrate to graphene flake inside, the I (G) and I (2D) should monotonously increases the reach a saturation value in graphene center. Meanwhile,

the ratio between I (2D) and I (G) remains two during the Raman mapping due to the single-layered feature. Differently, I (D) increases, reaches a maximum at edge area and then decreases. The maximum value is achieved when the incident laser spot crosses the edge. As shown in Fig.S3b, we fit the change of I (D) with Gauss method and I (G)/I (2D) with Boltzmann function. We define graphene edge where the maximum I (D) is, when I (G) and I (2D) are simultaneously half the saturation value. The position of graphene edge is shown in Fig.1c-h as red dashed lines.

3. The separation of mechanical strains from charge doping in graphene

As reported[3], there are two factors which can induce the shift of graphene Raman peaks: mechanical strains (compressive or tensile) and charge doping from substrate (Hole-doping generally on the silicon dioxide substrate). We can separate the mechanical strain from charge doping through Raman Characterization. The details of measurement can be found in the Ref. [3]. Here, we just give a brief introduction. As shown in Fig.S4, the red and blue solid lines represent the Raman peak shift under different stains or different density of holes (electrons), respectively. The green dot (denoted O) is obtained from a freestanding graphene which is not affected by strain or charge doping. The coordinate is (1581.6±0.2cm^{-1} 2676.9±0.7cm^{-1}). We separate the strain of graphene from the charge doping using decomposition method as follows. The vector OP=ae_T+be_H, e_T and e_H are unit vectors for tensile strain and hole doping effects. Hence, the distribution of tensile strain in graphene sample can be extracted from the coefficient ‘a’ and the hole-doping effects can be extracted from the parameter ‘b’. The ω_G-ω_{2D} space is divided into four quadrants including Q1, Q2, Q3 and Q4. Q4 is attributed to tensile strains and Q1 is attributed to compressive strains. The Q2 and Q3 are meaningless because that both hole and electron doping should induce the increase of $\omega_{G.}$ Our mechanically exfoliated graphene samples on the silicon dioxide substrate are mostly dominated by tensile strains.

4. Extraction of characteristic length scale of the disorder

In order to characterize the spatial components of random disorder, we conduct the two-dimensional fast Fourier transform (FFT) of strain maps, as shown in Fig. S5. When the Anderson transition occurs, the Fourier spectrum shows more and more widespread patterns, indicating that the disordered level of graphene system increases. The discrete distribution in the momentum space indicates that the disorder induced by strains is random, not periodic. In order to extract the characteristic length scale of disorders, we measure the size of every strain spot (green dashed circles in Fig.S6) and the distance between two neighboring spots (blue dashed lines in Fig. S6). For the weak and strong scattering condition, the average size of the strain spots is 263±101.4 nm and 304±170.3 nm, respectively. Given the fact that the plasmonic wavelength in our case is ~220 nm, the length scale of random disorders is in the same order of magnitude with mode wavelength. Meanwhile, the average distance between two neighboring spots is 667±227.3 nm for weak scattering and 674±100 nm for strong scattering, which are both in the same order of magnitude with plasmonic wavelength. The characteristic length scale with the same order of magnitude compared with plasmonic wavelength is essential for the observation of Anderson localization.

5. Determination of surface roughness of graphene flakes

The rough surface induces the enhanced plasmonic amplitude due to the formation of ‘Hot Spots’, which is the explanation for surface enhanced Raman spectroscopy[4,5]. This uneven distribution of plasmon cause an adverse impact when we study the Anderson localization. In conventional metal thin film, it is quite challenging to build an atomically flat surface with the limitation of preparation technologies. Hence, it is hardly distinguish the Anderson localization from the enhanced amplitude just caused by protuberances on surface[6].

The height of mechanically exfoliated graphene on substrate is less than 1.0 nm, which provides a relatively flat surface[7]. As shown in Fig.S7a-c, our graphene flakes are single-layered and with a height of 0.6 ~0.8nm. The different height is caused by different thickness of air layer between

graphene and substrate during sample preparations. We show the roughness of graphene surface in Fig.S7d-f as blue dots (extracted from corresponding blue dashed lines in Fig.S7a-c). No matter what the disorder level is, the roughness of graphene surface remains less than 0.2nm, which is very important for the study of Anderson localized plasmons.

6. Nano-infrared images of graphene plasmon with different localized state

We conduct optical nano-imaging of graphene plasmon with different localized status under different incident frequencies. The near-field amplitude is normalized by Si standard reference sample as following[8]:

$$s_4(\omega) = s_4^0(\omega)/s_4^{Si}(\omega)$$

Here, $s_4^0(\omega)$ and $s_4^{Si}(\omega)$ are the forth-order demodulated harmonics of the near-field amplitude probed for graphene flake and Si standard sample, respectively. In Fig.S8, we display the near-field amplitude of expanded plasmon. We observe longer plasmonic wavelength for lower incident frequency, according with well-known dispersion relation of graphene plasmon[8] (Fig.3d). The nano-images of weak localized and Anderson localized plasmons are shown in Fig.S9 and Fig.S10, respectively. The near-field images contain intrinsic properties of graphene plasmon, including wavelength (Fig.3 in main text), damping rate (Fig.S11), and dispersion (Fig.3g). All these intrinsic properties are independent of localized status of graphene plasmon, as mentioned in the main text. All nano-infrared images are collected at ambient atmosphere.

7. Fitting method of the damping rate of graphene plasmon

Based on the reported fitting method[9], we extract the damping rate of graphene plasmon with different localized states. The fitting details can be found in the ref. [8] and we just show a brief description here. We conduct average line scans of the complex ξ perpendicular to the graphene

edge and subtract the background through two dimensional Fast Fourier Transform (2D-FFT). The ξ could be fitted well with the formula:

$$\xi_{opt}(y) = A\frac{e^{i2q_py}}{\sqrt{y}} + B\frac{e^{iq_py}}{y}$$

The first section is tip-launched plasmon and the second one is edge-launched. The edge-launched graphene plasmon is very weak and can be neglected in our condition[8,10]. So, the Re (q) and Im (q) can be extracted. The γ_p can be calculated from Im(q)/Re(q) as a dimensionless figure of merit of propagation damping, as shown in Fig.S11. When fitting the plasmonic line-profiles, we exclude the first dominate fringe due to its complicated interference (edge-mode and sheet-mode) and tip-substrate coupling effect[9]. The extracted damping rates increase when the Anderson transition occurs.

8. Calculation of mean free paths of graphene plasmons

We calculated the mean free paths of graphene plasmon (l^*) based on scaling theory[11] as following:

$$\xi_{LOC} = l^* \exp(0.5\pi k_p l^*)$$

The extracted l^* in the weak localization (Tab.S1) is larger than Anderson localization (Tab.S2). Detailed explanation is provided in the main text. Localization length as a function of $k_p l^*$ allows us to directly determine the transition window for Anderson localization of graphene plasmons (Fig.4b).

9. Theoretical calculation of dispersion of graphene plasmons

In order to get the dispersion, we calculate the complex reflectivity $r_p\ (q,\ \omega)$ of graphene/SiO_2 structure. When we treat the system as a semi-infinite substrate, the reflectivity can be expressed as[12]:

$$r_p = \frac{\varepsilon_2 k_{1z} - \varepsilon_1 k_{2z} + k_{1z}k_{2z}\,\sigma(q,\omega)/(\varepsilon_0\omega)}{\varepsilon_2 k_{1z} + \varepsilon_1 k_{2z} + k_{1z}k_{2z}\,\sigma(q,\omega)/(\varepsilon_0\omega)}$$

where ε_0 is the vacuum permittivity, ε_1 and ε_2 are relative permittivity of air and SiO_2. ω is incident frequency and q is the plasmonic wavevector. The k_{1z} and k_{2z} represent the z-components of the wavevector of the incident and the transmitted plane-waves, respectively. The conductivity of graphene ($\sigma(q,\omega)$) is derived with random-phase-approximation (RPA). Extracted maximum imaginary part of r_p is shown as background color in Fig.3g.

10. The theory of Anderson localization in a 2D metal system

Theoretically, the distribution of polaritonic field in two-dimensional disordered metal system can be described by a set of Kirchhoff equations, whose detailed derivation can be found in Ref [6, 13-16]. Here, we represent the brief introduction as followings:

In a semi-continuous film composed with metal grains randomly distributed on a dielectric substrate, the local field $\mathrm{E(r)}$ can be represented as [6]:

$$\mathrm{E(r)} = -\nabla\phi(\mathrm{r}) + \mathrm{E_0(r)}$$

where $\mathrm{E_0(r)}$ is the applied field and $\phi(\mathrm{r})$ is the potential of the fluctuating field inside the film. The current density $\mathrm{j(r)}$ at the point r is given by Ohm's law:

$$\mathrm{j(r)} = \varepsilon(\mathrm{r})[-\nabla\phi(\mathrm{r}) + \mathrm{E_0(r)}]$$

By the combination of current conservation law, the equation can be described as:

$$\nabla\{\varepsilon(\mathrm{r})[-\nabla\phi(\mathrm{r}) + \mathrm{E_0(r)}]\} = 0$$

In order to calculate the local electric field in the system, we need to discretize the above equation on disordered lattice [6]. The partial differential equation is reduced to a set of Kirchhoff equations. The Kirchhoff equations are characterized by the Hamiltonian H [referred as the Kirchhoff

Hamiltonian (KH)] with off-diagonal and diagonal elements. The KH formally maps the Hamiltonian for the Anderson transition problems [13], with both on- and off-diagonal correlated disorder as [14]:

$$(\mathrm{H}' + \mathrm{i}\kappa\mathrm{H}'')\phi(\mathrm{r}) = \mathrm{E}$$

where H' and H'' are the real-part and imaginary-part of the Kirchhoff Hamiltonian, associated with lattice potential. $\kappa = \varepsilon''(\mathrm{r})/\varepsilon'(\mathrm{r})$. E is the localized electric field, which is proportional to the applied field $\mathrm{E_0(r)}$. The potential $\phi(\mathrm{r})$ can be expressed in terms of the eigenfunctions (φ_n) of H' as [6]:

$$\phi(\mathrm{r}) = \sum\nolimits_{\mathrm{n}} \mathrm{A_n}\varphi_{\mathrm{n}}$$

Then

$$(\mathrm{i}\kappa\mathrm{b} + \Lambda_{\mathrm{n}})\mathrm{A_n} + \mathrm{i}\kappa\sum\nolimits_{\mathrm{m}\neq\mathrm{n}}\langle\varphi_{\mathrm{n}}|\mathrm{H}''|\varphi_{\mathrm{m}}\rangle\mathrm{A_m} = \mathrm{E_n}$$

$$\mathrm{b} = \langle\varphi_{\mathrm{n}}|\mathrm{H}''|\varphi_{\mathrm{n}}\rangle\sim 1$$

$$\mathrm{E_n} = \langle\varphi_{\mathrm{n}}|\mathrm{E}\rangle\sim\mathrm{E_0a}$$

In the zeroth approximation, the local potential $\phi(\mathrm{r})$ is solved as

$$\phi(\mathrm{r}) = \sum\nolimits_{\mathrm{n}} \mathrm{E_n}\,\varphi_{\mathrm{n}}(\mathrm{r})/(\Lambda_{\mathrm{n}} + \mathrm{i}\kappa\mathrm{b})$$

It strongly fluctuates and the averaged field intensity can be described as:

$$<|\,\mathrm{E}\,|^2> \sim\langle|\nabla\phi(\mathrm{r})|^2\rangle\sim\mathrm{E_0^2}\int\frac{\rho(\Lambda)[\mathrm{a}/\xi(\Lambda)]^2}{\Lambda^2+\kappa^2}\mathrm{d}\Lambda$$

where, $\rho(\Lambda)$ is the dimensionless density of states for the H' and a is structure parameter. Therefore, the field distribution in the two-dimensional disordered metal film can be described as a set of the KH eigenfunctions localized within $\xi(\Lambda)$, with the enhanced amplitude of $E_{max}\sim E_0\kappa^{-1}(a/\xi)^2$ [6]. Although the calculation is conducted in the 2D metal film, the main conclusions including Anderson

localized field with one specific localization length and its enhanced amplitude are appropriate for graphene.

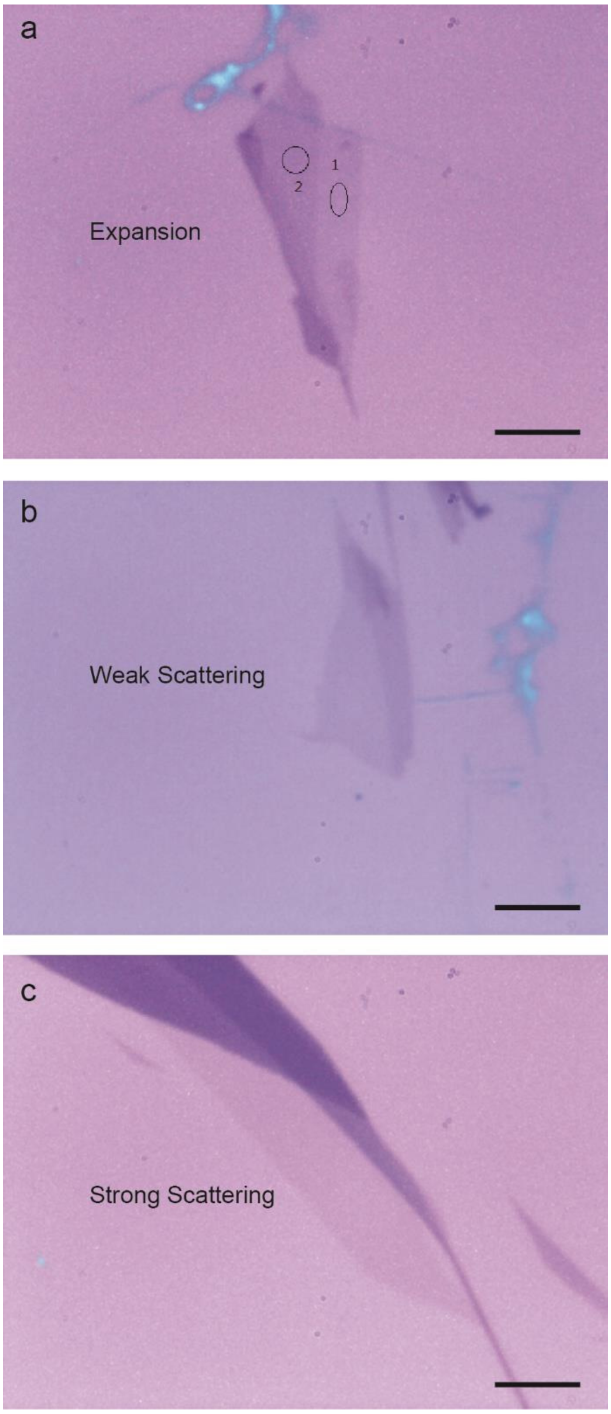

Figure S1. Optical micrograph of our graphene flakes. **(a-c)** Flakes with low, medium and high disordered level, respectively. The single-layered graphene can be easily discerned through optical contrast. This optical contrast measurement cannot determine the different strains distribution in graphene. Scale bar: 5μm

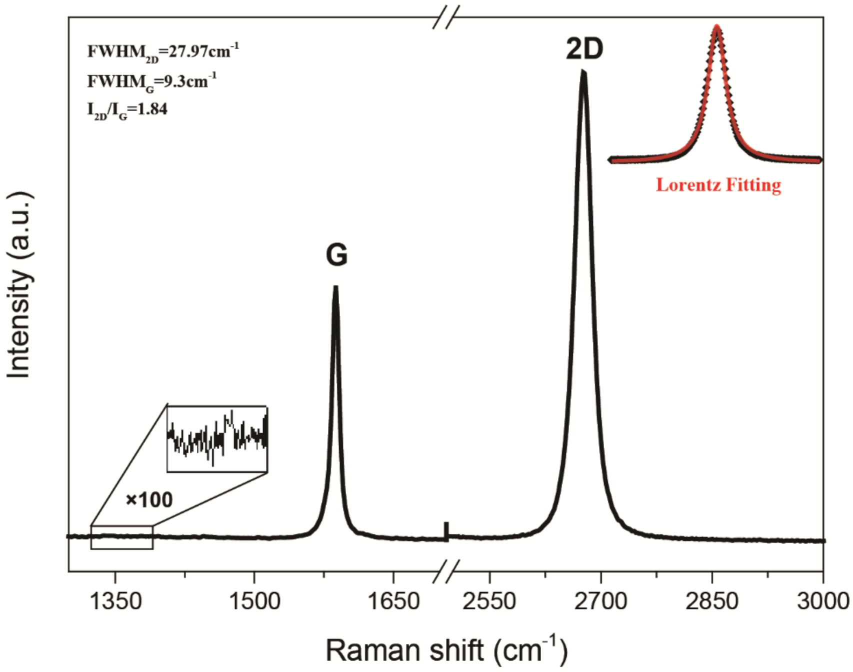

Figure S2. The representative Raman spectrum collected at central area of graphene flakes. All Raman responses including narrow spectral band of 2D and G peak, twofold ratio between 2D and G peak, great Lorentz fitting of 2D peak (shown in inset), and absence of D peak indicate that our mechanically exfoliated graphene is smooth and single-layered.

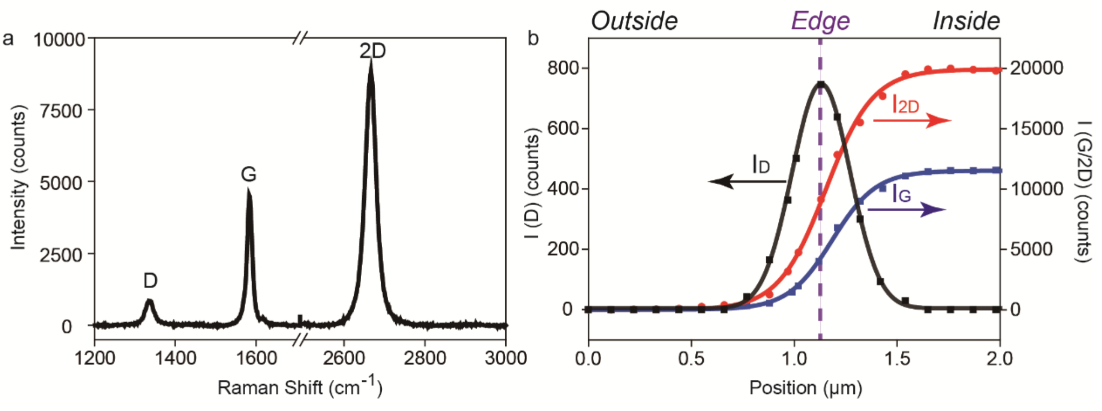

Figure S3. (a) Representative Raman spectrum collected at graphene edge. The D peak is observed at graphene edges, which act as one dimensional defects allowing elastic backscattering of electrons. **(b)** Line-profiles variation of the intensity of D (black), G (blue), 2D (red) peak from outside to inside graphene sample. 1D Raman line-mapping can determine the position of graphene edges (purple dashed line).

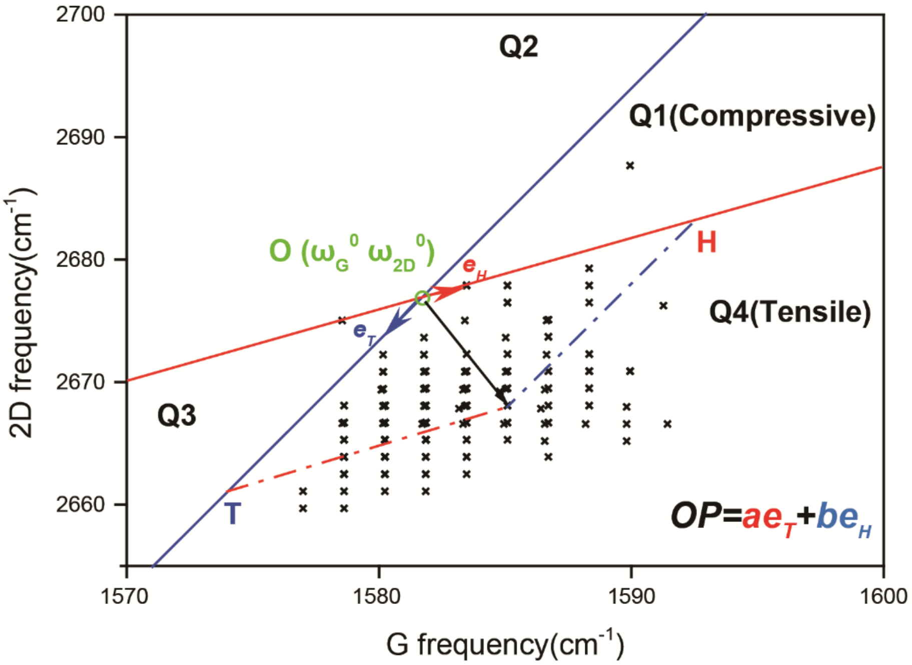

Figure S4. The extraction of strains and charge doping in graphene system by Raman measurements. We separate the strain of graphene from the charge doping using decomposition method.

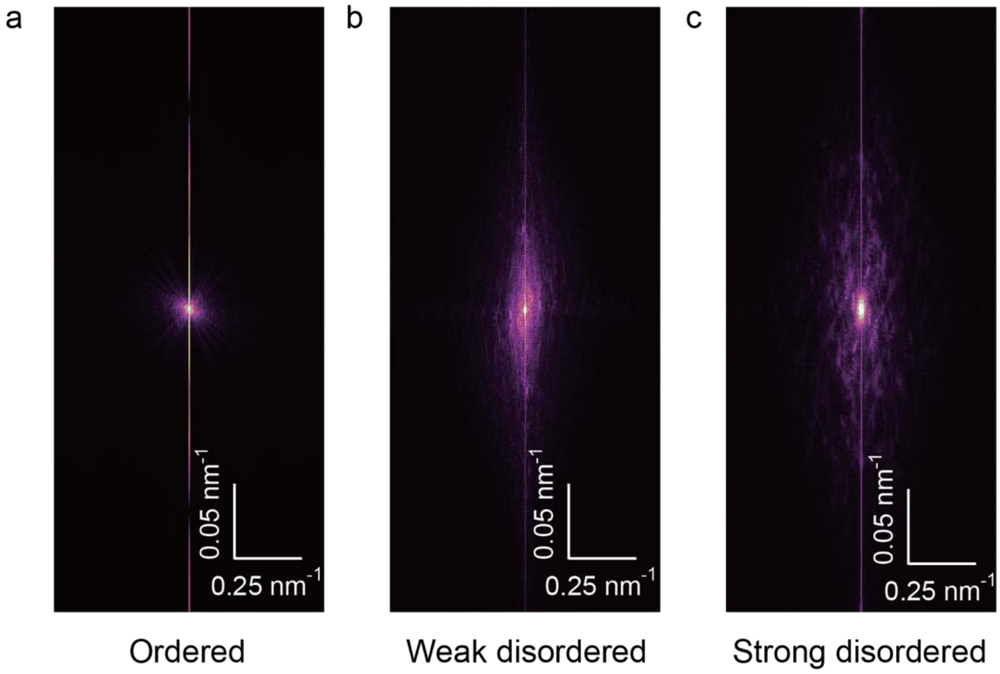

Figure S5. The two-dimensional Fast Fourier Transform (FFT) of the strain maps. **(a)** The FFT image of strain map in Fig. 1c, with low disordered level. **(b)** The FFT image of strain map in Fig.1d, with medium disordered level. **(c)** The FFT image of strain map in Fig. 1e, with high disordered level.

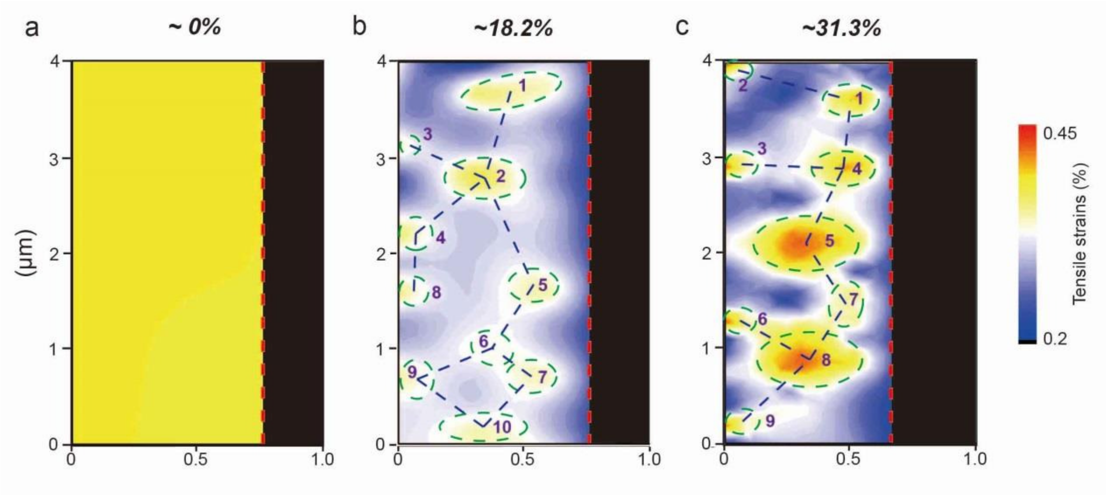

Figure S6. The extraction of characteristic length scale of random disorders. **(a-c)** The strain maps of low, medium and high disordered levels, which are also shown in Fig.1c-e. The green dashed circles represent the sizes of strain spots. The blue dashed lines represent the distance between two neighboring spots.

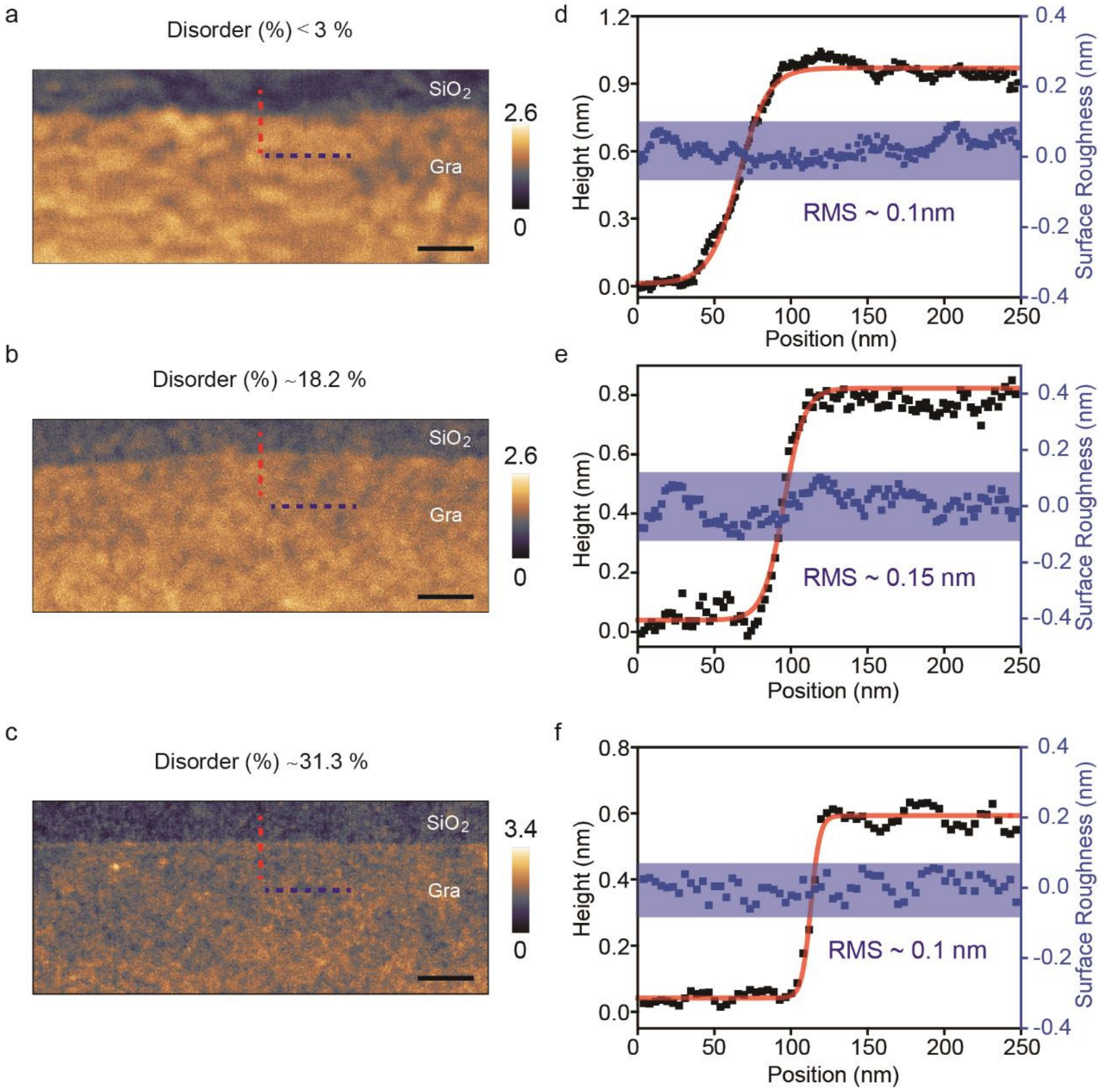

Figure S7. The surface topographical images of graphene with different disordered levels. **(a-c)** The AFM images of graphene with low, medium, high disordered level, respectively. These images are collected simultaneously with near-field optical images shown in Fig.2. The darker area is SiO_2 substrate and brighter area represents graphene. Scale bar is 300 nm. **(d-f)** The corresponding AFM line-profiles along the red dashed lines in (a-c), respectively. In order to determine the roughness of graphene surface, we also show the line-profiles along blue dashed lines. The Root-Mean-Square (RMS) of surface roughness shows similar value (~0.1 nm) in all disordered systems.

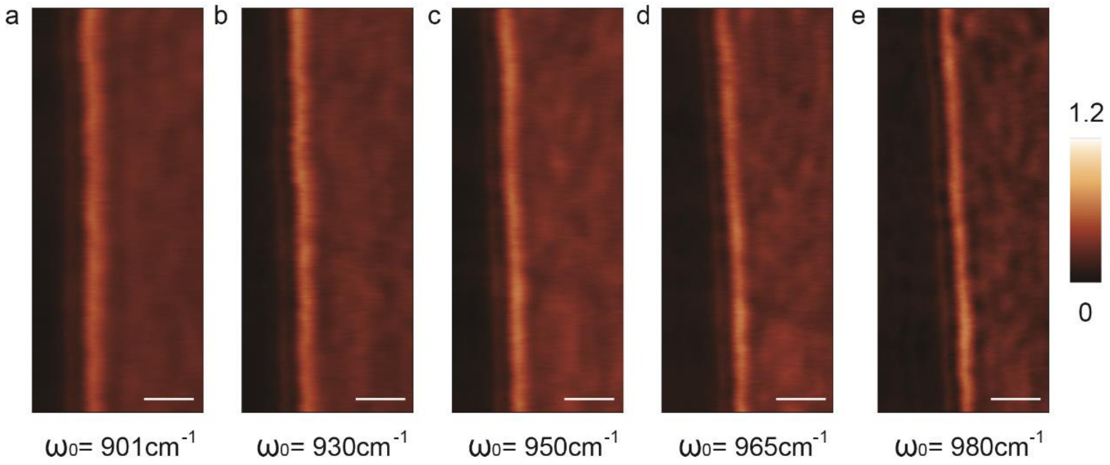

Figure S8. Nano-infrared images of graphene plasmons with expansion state under incident frequency of 901cm^{-1} (a), 930 cm^{-1} (b), 950 cm^{-1} (c), 965 cm^{-1} (d), and 980 cm^{-1} (e), respectively. The scale bar is 300 nm.

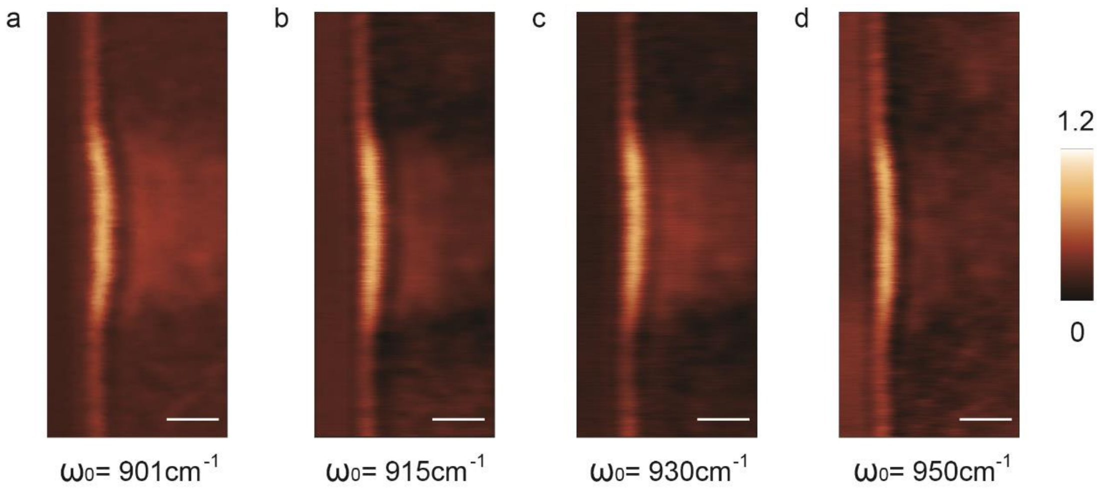

Figure S9 Nano-infrared images of graphene plasmons with weak localization state under incident frequency of 901cm^{-1} (a), 915 cm^{-1} (b), 930 cm^{-1} (c), and 950 cm^{-1} (d), respectively. The scale bar is 300 nm.

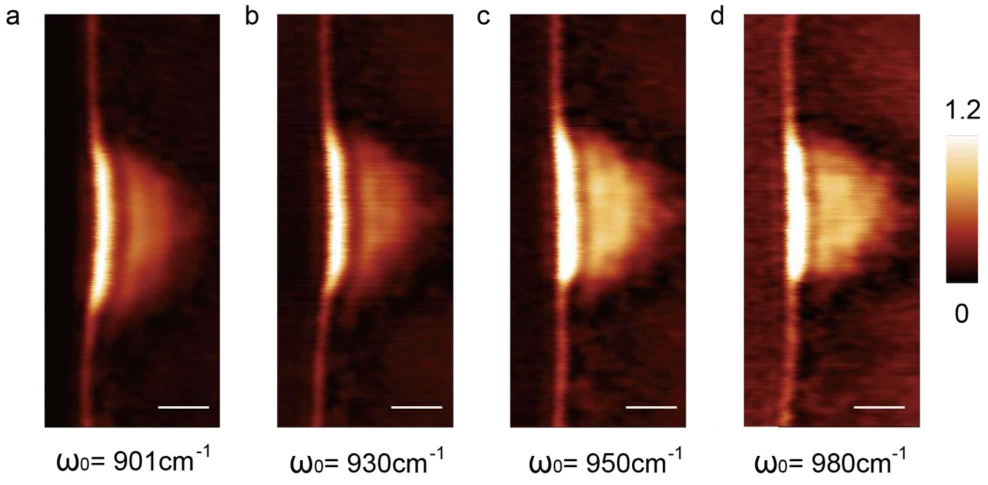

Figure S10. Full-sized nano-infrared images of graphene plasmons with Anderson localization state under incident frequency of 901cm^{-1} (a), 930 cm^{-1} (b), 950 cm^{-1} (c), and 980 cm^{-1} (d), respectively. The scale bar is 300 nm.

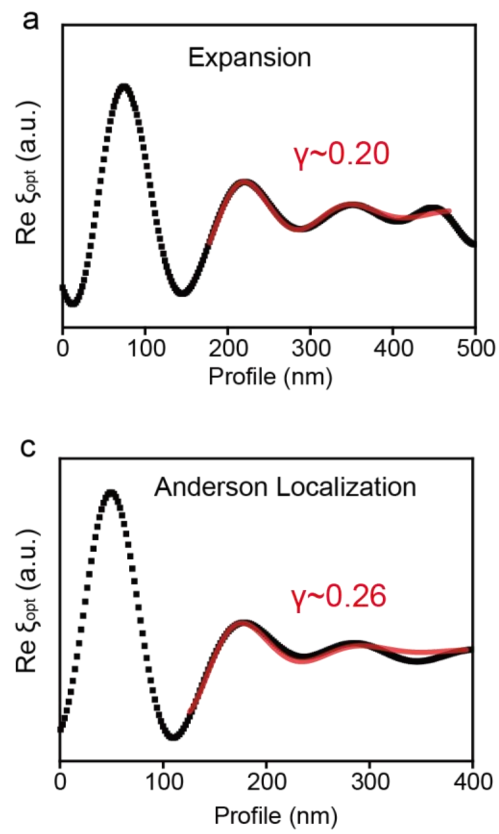

Figure S11. The extraction of plasmonic damping in expansion (a), weak localization (b), and Anderson localization (c) condition, respectively. The black squares show experimental near-field amplitude, with a subtraction of background through 2D-FFT. The experimental results are fitted through sine-damping oscillation function (red lines). We exclude the first fringes for fitting due to the complicated interference between edge mode and sheet mode and tip-substrate coupling effect.

ω_0	901cm^{-1}	915cm^{-1}	930cm^{-1}	950cm^{-1}
l^* (nm)	50.8282	49.7183	47.1076	42.7326
$k_p l^*$	1.2707	1.2927	1.3190	1.3674

Table S1. The scaling-theory extracted mean free paths (l^*) of graphene plasmons and scattering strength ($k_p l^*$) in weak localization condition under different incident frequencies.

ω_0		901 cm^{-1}	930 cm^{-1}	950 cm^{-1}	980 cm^{-1}
Anderson localized mode	l* (nm)	44.1677	43.7203	39.6072	36.6361
	$k_p l$*	1.0972	1.1784	1.1699	1.2097

Table S2. The scaling-theory extracted mean free paths (l*) of graphene plasmons and scattering strength ($k_p l$*) in Anderson localization condition under different incident frequencies.

References

[1] A. C. Ferrari, J. Meyer, V. Scardaci, C. Casiraghi, M. Lazzeri, F. Mauri, S. Piscanec, D. Jiang, K. S. Novoselov, and S. Roth, Physical Review Letters **97**, 187401 (2006).

[2] C. Casiraghi, A. Hartschuh, H. Qian, S. Piscanec, C. Georgi, A. Fasoli, K. S. Novoselov, D. M. Basko, and A. C. Ferrari, Nano Letters **9**, 1433 (2009).

[3] J. E. Lee, G. Ahn, J. Shim, Y. S. Lee, and S. Ryu, Nature Communications **3**, 1024 (2012).

[4] P. L. Stiles, J. A. Dieringer, N. C. Shah, and R. P. Van Duyne, Analytical Chemistry **77** (2008).

[5] Z. Fei, J. J. Foley, W. Gannett, M. Liu, S. Dai, G. Ni, A. Zettl, M. M. Fogler, G. P. Wiederrecht, and S. K. Gray, Nano Letters **16**, 7842 (2016).

[6] S. Grésillon, L. Aigouy, A. Boccara, J. Rivoal, X. Quelin, C. Desmarest, P. Gadenne, V. Shubin, A. Sarychev, and V. M. Shalaev, Physical Review Letters **82**, 4520 (1999).

[7] A. K. Geim and K. S. Novoselov, Nature Materials **6**, 183 (2007).

[8] J. Chen, M. Badioli, P. Alonso-González, S. Thongrattanasiri, F. Huth, J. Osmond, M. Spasenović, A. Centeno, A. Pesquera, and P. Godignon, Nature **487**, 77 (2012).

[9] A. Woessner, M. B. Lundeberg, Y. Gao, A. Principi, P. Alonsogonzalez, M. Carrega, K. Watanabe, T. Taniguchi, G. Vignale, and M. Polini, Nature Materials **14**, 421 (2015).

[10] Z. Fei, A. S. Rodin, G. O. Andreev, W. Bao, A. S. Mcleod, M. Wagner, L. Zhang, Z. Zhao, M. H. Thiemens, and G. Dominguez, Nature **487**, 82 (2012).

[11] T. Sperling, W. Buhrer, C. M. Aegerter, and G. Maret, Nature Photonics **7**, 48 (2013).

[12] L. Novotny and B. Hecht, Physics Today **60**, 62 (2007).

[13] B. Kramer and A. Mackinnon, Reports on Progress in Physics 56, 1469 (1993).

[14] V. M. Shalaev and A. K. Sarychev, Physical Review B 57, 13265 (1998).

[15] F. Brouers, S. Blacher, and A. K. Sarychev, Physical Review B 58, 15897 (1998).

[16] F. Brouers, S. Blacher, A. Lagarkov, A. K. Sarychev, P. Gadenne, and V. M. Shalaev, Physical Review B 55, 13234 (1997).
